# Co-Production Performance Evaluation in Healthcare. A Systematic Review of Methods, Tools and Metrics

**DOI:** 10.3390/ijerph18073336

**Published:** 2021-03-24

**Authors:** Marta Marsilio, Floriana Fusco, Eleonora Gheduzzi, Chiara Guglielmetti

**Affiliations:** 1 Department of Economics, Management and Quantitative Methods (DEMM), Università degli Studi di Milano, via Conservatorio, 7, 20122 Milan, Italy; floriana.fusco@unimi.it (F.F.); chiara.guglielmetti@unimi.it (C.G.); 2 School of Management, Politecnico di Milano, via Lambruschini 4, 20156 Milan, Italy; eleonora.gheduzzi@polimi.it

**Keywords:** co-production, co-creation, performance, evaluation, measure, outcome, performance evaluation system, healthcare

## Abstract

Co-produced practices and publications in the healthcare sector are gaining momentum, since they can be a useful tool in addressing the sustainability and resilience challenges of health systems. However, the investigation of positive and, mainly, negative outcomes is still confused and fragmented, and above all, a comprehensive knowledge of the metrics used to assess these outcomes is lacking. To fill this gap, this study aims to systematically review the extant literature to map the methods, tools and metrics used to empirically evaluate co-production in health services. The search took place in six databases: Scopus, Web of Science, Psych INFO, PubMed, Cochrane and CINAHL. A total of 2311 articles were screened and 203 articles were included in the analysis, according to PRISMA guidelines. Findings show that outcomes are mainly investigated through qualitative methods and from the lay actor or provider perspective. Moreover, the detailed categorisation of the quantitative measures found offers a multidimensional performance measurement system and highlights the impact areas where research is needed to develop and test new measures. Findings should also promote improvements in empirical data collection on the multiple faceted co-produced activities and spur the consciousness of the adoption of sustainable co-productive initiatives.

## 1. Introduction

Co-production is widely considered a promising tool for dealing with current challenges in the health sector [1,2,3], where resources are being significantly reduced. Conversely, patients’ expectations of higher-quality services are growing amid increasing demand caused by an aging population and the rise in chronic diseases. This pressures healthcare systems, challenging their long-term sustainability. To this end, policy makers (e.g., [4]) have promoted the development of more patient-centred personalised care based on new relational models, in which patients, their informal caregivers and local communities share responsibilities with care providers, thus enabling them to feel part of a team and fostering the quality of services. Patients are asked to participate actively and act as consumer producers, next to and in interaction with healthcare professionals and other stakeholders in healthcare, such as health providers, general practitioners, social services etc. [5,6]. This approach calls for a “community-accountable health development system” that can coordinate stakeholders’ interests [7]. These aspects also assume relevance for unexpected challenges like rare events and pandemics. In recent months, the need to revise existing healthcare and social care systems with broader perspectives considering the skills, competences and experiences of whole ecosystems has appeared even more crucial due to the COVID-19 outbreak and the consequent risks [8,9,10]. Co-production has been identified as one possible solution to manage COVID-19 and to deliver public services enhancing community resilience through self-helping neighbourhood and volunteering activities. The collaboration of citizens (such as self-quarantining or wearing masks) in the face of limited opportunities for enforcement or encouragement guaranteed the success of the measures adopted by the health system in dealing with increasing infections [11].

Involving different stakeholders and relying on their cooperation and resource integration, co-production accentuates the complex and adaptive nature of the health service system, posing some challenges but also extending opportunities [12,13]. One of the challenges that certainly arises is to evolve a performance management and evaluation systems capable of systematically considering diverse value perspectives that come into play [14]. In this regard, it must be emphasized that there is increasing interest in understanding the benefits of co-production for the healthcare ecosystem [15]. Extant knowledge shows that co-production positively impacts on specific single dimensions, e.g., provider cost-efficiency (e.g., [16,17]); health outcomes (e.g., [18]); perceived service quality (e.g., [19]); service accessibility (e.g., [20]); customer satisfaction and quality of life (e.g., [21,22]); and compliance (e.g., [23]). Nevertheless, a systematic evaluation of the impacts of co-production on the different stakeholders involved and its sustainability over time still is lacking [24,25]. This could also be ascribed to the “magic” nature of the co-concepts and the normative assumptions behind them, according to which a co-produced service ought to be a “better service” [2,26]. Moreover, an outcomes evaluation could be performed with different methods, indicators and timeline. Some recent reviews show that often the co-production effects are limited to narrative case studies, studies with small sample sizes and with poor attention to their sustainability [25,27]. Consequently, a comprehensive and robust co-production performance evaluation system would support in identifying the most suitable research approaches, methods and metrics for each specific outcome. This would also help to question the “celebratory” nature of co-production and to advance the investigation of unsuccessful and negative cases by challenging the “enchanting” nature of co-concepts and their effectiveness [26,28].

This paper aims to fill this gap. Drawing from the recent systematic categorisations of co-production outcomes according to all actors involved (lay actors, regular service providers and communities) [29], it systematically reviews the current literature to identify the methods, tools and metrics used to evaluate health co-produced services, according to each stakeholder. The paper offers a blueprint multidimensional performance measurement system that factors in the values of the multiple stakeholders and highlights the impact areas where further research is needed to develop and test new measures. The findings could promote improvements in empirical data collection on multiple faceted co-produced activities and spur consciousness of the adoption of sustainable patient-based initiatives. 

The structure of this article is organised as follows. First, the research strategy adopted to conduct the review is presented. Subsequently, the main findings are reported and discussed. Finally, the paper provides recommendations and guidelines for future studies on co-production in the public and healthcare sectors.

## 2. Materials and Methods

To perform a replicable and transparent systematic review analysis, the guidelines of the Preferred Reporting Items for Systematic Reviews and Meta-Analyses (PRISMA) have been adopted for the paper selection phase [30,31]. A PRISMA statement is an evidence-based minimum set of items for reporting systematic reviews and meta-analyses, useful for ensuring the rigor of systematic searches and that all relevant literature is included to decrease selection bias. The eligibility criteria and the four-phase flow of selection process are detailed below.

### 2.1. Study Eligibility Criteria

The objective of this study is to assess which research methods, tools and metrics have been adopted in the extant literature to measure the effects of co-production in the healthcare and public sectors. PRISMA guidelines distinguish between study eligibility criteria and report eligibility criteria. The first criterion concerns the characteristics of the study that allow its inclusion or exclusion.

- *Topic*: Included studies must be focused on evaluating the effects of co-production. Moving from Nabatchi et al.’s (2017) [32] co-production definition, in this study we include a wide variety of activities in which service providers (i.e., healthcare providers) and lay actors (i.e., patients and/or carers) voluntarily work together in any phase of the health service cycle (i.e., commissioning, design, delivery, and assessment). As the concepts of “co-production” or “co-creation” are strictly related [33,34] and given their essential similarity [35], the systematic review includes both the literature on co-production and on co-creation. Considering both concepts adds useful insights to the outcome evaluations.
- *Aim of the research*: Only empirical studies targeting co-production/co-creation outcomes are included. Therefore, conceptual papers, reviews and protocols are excluded. No limitations on study design were introduced.
- *Field*: Both the health and public sector co-production literature were investigated. The second stream has been considered well-developed [3], and recent reviews on co-production in public show that health services emerge as one of the main sectors of interest [29]. Moreover, co-production outcome evaluation in public services (with a particular emphasis on health) has recently gained the main attention of distinguished scholars in the field [15,36]. Hence, this multidisciplinary approach can provide valuable insights into health sector co-production, with the caveat of considering contextual distinctions between the two sectors.

### 2.2. Search Strategy and Report Eligibility Criteria

Data were extracted from the largest peer-reviewed research literature databases in both fields (Scopus, Web of Science, Psych INFO, PubMed, Cochrane and CINAHL) in November 2020. The query was shaped with a three-level structure: the first defines phenomena; the second, the context; and the third, the unit of analysis. The choice of keyword limits was accurate for ensuring the completeness of the research, but also for focusing on the topic [37]. As displayed in Table 1, two queries were launched, respectively, concerning the health or public sector context, searching keywords in the string “topic” or “abstract, title and keywords”, according to the specific tag used by each database. 

Moreover, the following report eligibility criteria were selected:

- *Language*: Only English records are included, since it is the most-used language in the scientific literature and due to practical difficulties of translation.
- *Type of studies*: The review considers only articles published in scientific and peer-reviewed journals to ensure the reliability of sources and results.
- Publication status: Both published articles and, if available, articles in press were included to embrace the largest number of publications existing at the date of the search.
- *Year of publication*: No filters are adopted on the years of publication.

This strategy allowed the identification of 4192 records in the healthcare context and 492 in the public context. 

**Figure 1:**
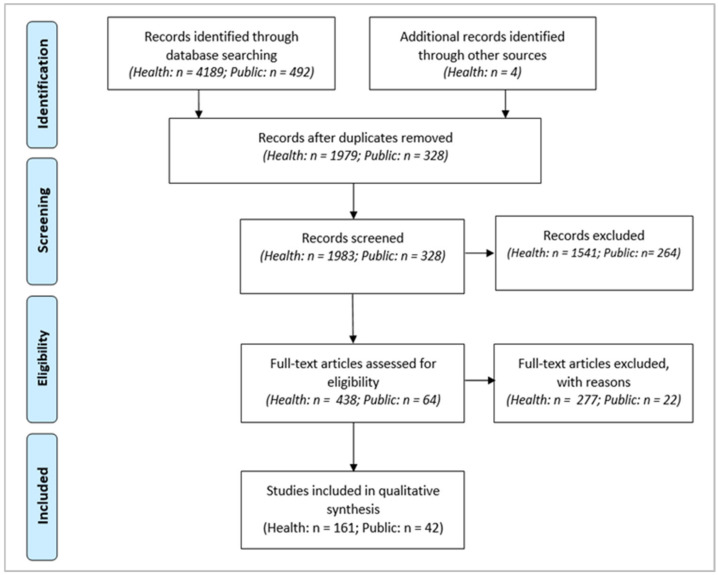
PRISMA flowchart.

Figure 1 shows the PRISMA flowchart used to map the selection procedure.

After record identification and duplicate removal, the screening phase was manually conducted on titles and abstracts. In the health dataset, 438 articles were considered eligible for full-text assessment, including 4 articles added with the snowball technique. In the public dataset, 64 articles were considered eligible for full-text assessment. In the second step of the screening phase, in the health dataset, 277 articles were excluded with reasons (e.g., no evidence for co-production/co-creation effects, no patient or caregiver inclusion in processes), and 161 articles were included in the qualitative analysis. In the public dataset, 42 were included in the qualitative analysis, while 22 were excluded. Among those 22 excluded, 8 focused on the health context and were already included in the healthcare dataset. In each of the two phases, first, all authors separately screened a sample of articles to verify the level of accordance in eligibility assessment; then, the second and third authors selected the included articles. All authors approved the final dataset, which consisted of 203 papers. Disagreements were resolved through discussion and consensus. 

### 2.3. Data Collection

First, baseline information was extracted: author/s; title; journal; year; research questions; methodological approach (qualitative/quantitative/mixed); study design (e.g., case study, randomised controlled trial etc.); country of the study; field of the study (health/public) and context (e.g., mental health, primary care etc.).

For a detailed examination of the methods, tools and metrics used, articles were initially coded through Cepiku et al.’s (2020) outcomes identification and classification framework [29]. Specifically, within a comprehensive framework of co-production activation, management and evaluation for public services, they classified outcomes according to the actors affected by co-production: lay actors, service providers and the community at large. Considering the peculiarities of the health sector [27,38], a specific actor category was added to include outcomes for professionals, whose role and involvement in the co-production of the health service assume a specific role that must be distinctly analysed [27,39]. The focus on healthcare workers as a necessary part of the relationship between organisational processes and quality of care is not new in the literature [40]. Parkinson’s (2018) systematic review stressed the importance of the health of workers and their families as a competitive advantage as healthcare organisations strive to deliver resource-efficient, high-quality care to patients [41]. 

**Figure 2:**
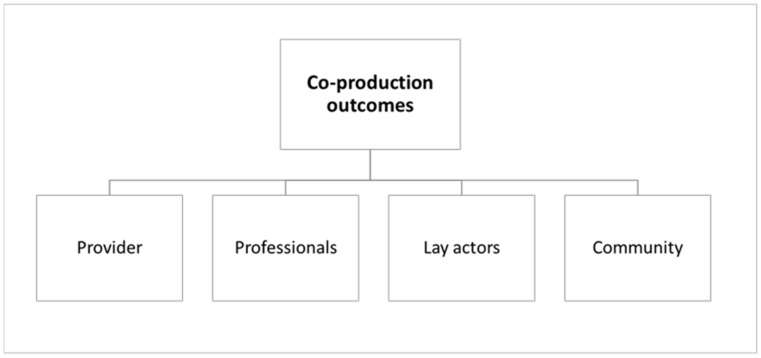
Outcomes’ coding framework.

Figure 2 represents the outcomes coding framework used to analyse the dataset.

## 3. Results

### 3.1. Article Overview

**Figure 3:**
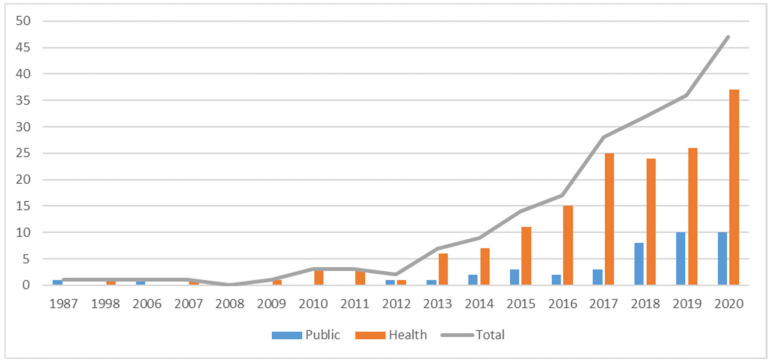
Annual scientific production—health and public.

The 203 articles included in the database cover the period from 1987 to November 2020. Figure 3 depicts the publication trends. The interest in the public field dates back to 1987, while in healthcare, the first article meeting the selection criteria is dated 1998. Attention to the co-production concept, both in the public and in healthcare fields, is growing exponentially: 69.5% of healthcare and 73.8% of public sector studies have been published in the last four years.

Considering journals, high fragmentation regarding articles per journal characterised both sub-datasets. Healthcare articles were published in 96 journals, of which only 27 (28.4%) have published at least two papers and only 18 have published at least three papers (11.2%). Research addressing the public sector has been published in 35 journals, of which only three (7.1%) had at least two articles (see Supplementary Materials Table S1).

Empirical evidence has been assessed in several countries, proving the adaptability and compatibility of these concepts within different contexts [3]. A strong geographical concentration is still detectable: 73% of health studies and 62% of public sector studies refer to five countries. This trend appears to be greater within the health field, in which almost 50% of the studies analysed are implemented in the UK (see Supplementary Materials Table S2). Considering the context of the research in the public domain, health and social care has the highest number of papers (11), followed by education (10) and general government (8), see Supplementary Materials Table S3. This result confirmed our methodological choice to include the public domain in the analysis as informative for healthcare investigation. Looking deeper into the healthcare domain, 61% of the papers deal with chronic or long-term disease patients. Another recurrent context is public health, especially concerning health prevention and promotion and the reduction of health inequalities in access to care. Increasing interest in the use of e-health and assistive technology is evident across all contexts (Table 2).

### 3.2. Performance Evaluation System: Research Design, Approach Methods and Tools

The articles selected for review were analysed to assess which research methods, tools and measures have been used by authors to evaluate the impacts of co-production in the healthcare and the public sectors. 

First, the research design and approach were considered. The adopted research design is clearly stated in a scant number of works. A full reading of the papers identified 34 with a longitudinal design, 8 with a cross-sectional design and 10 with a mixed design. 

As depicted in Table 3, about half of the reviewed articles adopted a qualitative approach, while the other half was equally divided between quantitative and mixed approaches. There are some distinctions between the two domains regarding quantitative and qualitative percentages, but they may not be considered remarkable due to the varied sizes of datasets.

Second, considering the number of stakeholders involved in the evaluation analysis, 47.8% of papers assessed the impacts only for one stakeholder, while 52.2% assessed the impacts for two or more stakeholders (Table 4). This evidence is common to both the health and public domains.

Third, to understand which are the main stakeholders considered in the co-production evaluation analysed and which are the methods of data analysis adopted, for each paper of the dataset, stakeholder outcomes (i.e., outcome for provider, outcome for professional, outcome for lay actors, outcomes for community) were identified and investigated. In total, 348 stakeholder outcomes occurred, 274 in the health domain and 74 in the public domain. On average, each paper reports impacts for 1.7 stakeholders. The results are summarized in Table 5.

The most evaluated impacts concerned the lay actors (41%) and the provider (35%), while the impacts on professionals and on the community are largely under-investigated. 

Considering the method of data analysis, there is a prevalent adoption of qualitative methods for all clusters of outcomes; however, some distinctions need to be highlighted. The evaluation of the outcomes for providers and lay actors is characterised by a high presence of quantitative analysis methods, whereas the outcomes for professionals and for a community have been assessed mainly through qualitative methods. Overall, the use of a quantitative approach is relatively more frequent in the healthcare domain. 

Quantitative data analysis methods include statistical analyses, such as structural equation modelling (e.g., [42,43,44]), factor analyses (e.g., [44,45]), correlation and regression analyses (e.g., [6,16,46]), analysis of variance (ANOVA or MANOVA) (e.g., [47]), nonparametric test (e.g., [48,49,50]), multiple imputation techniques (e.g., [51]), and cost analysis, such as cost minimisation analysis (e.g., [16]) and cost opportunity analysis (e.g., [17]). Qualitative data analyses mainly embrace content analysis (e.g., [52]) and deductive or inductive thematic analysis (e.g., [53,54,55]), including specific inductive methodology, such as Grounded Theory (e.g., [56,57,58]), Gioia methodology (e.g., [59,60]) and interpretative phenomenological analysis (e.g., [61]). 

Finally, tools used in co-production evaluation were investigated. Quantitative data collection tools consist of surveys, questionnaires and secondary data, e.g., archival administrative data. Qualitative data collection tools included surveys, questionnaires, in-depth or semi-structured interviews, focus groups, meetings, workshops and observations. It should be emphasised that 16 papers adopted a narrative approach or unspecified quantitative and/or qualitative data collection and analysis methods.

### 3.3. Co-Production Outcomes: Quantitative Measures and Metrics

**Figure 4:**
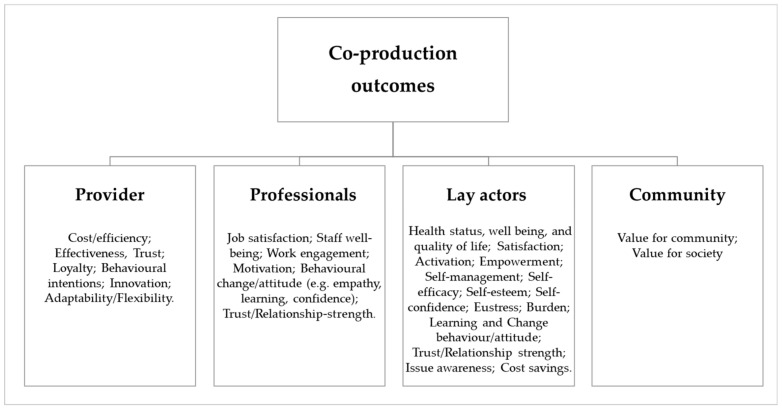
Analytical components framework.

The focus was then narrowed to the quantitative measures and metrics used in quantitative or mixed papers for assessing the effects of co-production. More precisely, for each stakeholder (i.e., providers, professionals, lay actors, the community), diverse outcome analytical components were identified and the type of measures/metrics specified. Specifically, the paper moves from the analytical components of outcomes identified for each actor (e.g., for lay actor enjoyment and satisfaction, empowerment etc.; for provider efficiency, effectiveness etc.; for the community value for society etc.) as in the work of Cepiku et al.’s 2020 [29]. Considering the peculiarities of the health sector [38], some adaptations resulted in the analytical components’ classification; Figure 4 shows the framework with the analytical components identified.

The measure and metrics have been grouped according to the following categories: international validated scales, other tools (i.e., other reliable tools, whose robustness has been statistically tested in a paper or recognised in a specific field; adapted instruments from other works; and ad hoc instruments that include single or group of items created ad hoc for a study) and single indicators (i.e., specific measures adopted as a proxy to evaluate impacts). 

Tables 6–9 summarise the results, proving analytical references for each component, measure or metric.

The following sections discuss the main results for the healthcare sector, highlighting the public literature when providing valuable additions.

#### 3.3.1. Outcomes for Provider

Five main analytical components of outcomes for providers have been measured: (i) cost-efficiency; (ii) effectiveness; (ii) trust; (iv) loyalty; (v) behavioural intentions; (vi) innovation and (vii) adaptability/flexibility (Table 6).

*Cost-efficiency* (productivity) is considered the relationship between the costs of input and the related output [29]. This category includes cost saving and other efficiency outcomes. Cost savings have mainly been assessed through cost–benefit analysis. For instance, Span et al. (2018) developed a cost-minimisation analysis to calculate the total cost for hospital-based and home-based health strategies [16]. Medical (i.e., drugs and visits) and non-medical costs (transportation costs and work/school days missed) have been considered. Other studies used ad hoc indicators to calculate cost savings (e.g., [62]). *Efficiency* outcomes have been measured through indicators, such as the reductions for occupied bed days and admissions (e.g., [63]). 

*Effectiveness* is the most assessed dimension for provider outcomes. It can be defined as the capacity to achieve the planned results in providing services. The effectiveness dimensions identified are service improvement, feasibility/acceptability of interventions or tools, and the usability of e-health tools. Service improvement has been evaluated mainly regarding increased accessibility or utilisation of a service through the use of specific indicators (e.g., [64,65]). This category assumes a particular meaning in healthcare, where the service is primarily effective when it generates an improvement or at least does not worsen the patient’s health. However, for classification purposes, these types of outcomes (i.e., health status, quality of life and well-being) have been considered adopting the lay actors’ perspective, given that they are the main beneficiaries of the service. In assessing service improvement, public sector findings go beyond the mere analysis of the increasing number of service users; they also assessed other features of the service, such as its frequency, distance from users [64] and performance (e.g., average examination course score) [66,67]. Several papers evaluated the usability of co-designing e-health tools through the System Usability Scale (SUS, [68]) (e.g., [69]); the Patient Education Materials Assessment Tool (PEMAT, [70]) has served to check the comprehensibility and actionability of health education material co-created by expert clinicians and patients in Badiu et al. (2017) [71]. Within effectiveness indicators, the percentage of patients declining (or accepting) an intervention (e.g., health screening) can be found [72].

*Trust* and *loyalty* are assessed mainly through ad hoc questionnaires, resulting from the previous literature (e.g., [73,74]). 

*Behavioural intentions*, such as recommendation intentions and positive word of mouth, have also been evaluated through other existing and validated tools, such as the behavioural intentions construct of Dagger et al. (2007), for example [75]. 

Few measures have been found on *innovation*, measured by Sehgal and Gupta (2020) with scale items created ad hoc, according to the extant literature.

*Adaptability and flexibility* of services, investigated concerning the decentralisation of power and higher response to users’ need in Dhirathiti (2019) [76] and McAllister et al. (2018) [77], have been measured, respectively, with ad hoc items and a validated scale. While these dimensions in the health sector have been mainly studied with micro and meso perspectives investigating changes at the patient–professional and provider levels, in the public sector, the analysis shifts at the government macro level by investigating the reorganisation of the governmental structure regarding the decentralisation of power [76].

#### 3.3.2. Outcomes for Professionals

The main outcomes of the analytical components concern: (i) job satisfaction; (ii) staff well-being; (ii) work engagement; (iv) motivation; (v) behavioural change; and (vi) trust in professionals/relationship strength (Table 7). 

*Job satisfaction* is a widely investigated construct in the academic literature, with many definitions and available operationalisations. However, it seems to be scarcely measured as a co-production outcome. Only few works addressed this issue, with confounding results. Three of four studies found that the co-creation of care increases the job satisfaction of mental health professionals [6,88,89], while Den Boer et al. (2017) did not find any correlation [46]. Two papers adopted the Measurement of Job Satisfaction (MJS, [90]) as one of the most reliable, valid and multidimensional measures of job satisfaction, while the other two use ad hoc tools.

*Job well-being* concerns the holistic perspective of workers’ physical and emotional status concerning their work environment. Levels of well-being have been assessed in some studies through the Social Production Function Instrument (SPF-IL scale, [91]), a 15-item validated scale including some relevant dimensions of subjective well-being (universal goals, affection, behavioural confirmation, status, comfort and stimulation). Burnout is measured through the most-known tool, the Maslach Burnout Inventory (MBI, [92]). Finamore et al. (2020) applied the MBI to verify the effect of co-produced personality disorder training on staff [93]. The same scale was used in Farnese et al. (2020) to investigate the effects of informal co-production between professionals and caregivers, but no correlation was found [94]. 

*Work engagement* and *motivation* are intended as active involvement and the willingness to perform one’s job. To verify the effect of co-delivery training on professionals, Hastings et al. (2018) used the Staff Positive Contributions Questionnaire (SPCQ, [95]) to evaluate the impact on staff motivation. The well-known Utrecht Work Engagement Scale (UWES, [96]) was used to measure work engagement in Ding et al. (2019) [6], which aimed at verifying the effect of patient participation in value co-creation with hospital nurses. 

*Behavioural change* considers all changes in skills and personal behaviour in clinical routines, including ways of relating to patients. Within this category, the dimension most assessed has been staff empathy and attitudes towards patients. They have been assessed mainly in studies focusing on co-delivery training for mental health staff. Among the validated scales adopted, Staff Empathy for people with Challenging Behaviour Questionnaire (SECBQ, [97]) can be mentioned, as well as Borderline Personality Disorder-Cognitive/emotional Attitudes Inventory (BPD-CAI/FAI, [98]). Self-efficacy, defined as the belief in one’s ability to succeed in specific situations or accomplish a task, has been measured through other reliable tools and adapted instruments.

*Trust* is considered related to this stakeholder, as the relationship between lay actors and professionals (e.g., dyadic relationship such as patient–doctor). Findings show that it has been assessed only with ad hoc or adapted instruments (e.g., [73,99]). For this outcome dimension, public metrics assessed are limited in number and do not add any valuable and innovative insights to the health findings. 

No specific indicators have been found for this stakeholder outcome. 

#### 3.3.3. Outcomes for Lay-Actor

Outcomes of co-production on lay actors are the most investigated with quantitative approaches. Fifteen analytical components have been identified: (i) health status; (ii) satisfaction; (ii) activation; (iv) empowerment; (v) self-management; (vi) self-efficacy; (vii) self-esteem; (vii) self-confidence; (ix) eustress; (x) burden; (xi) learning; (xii) changes in behaviour/attitude; (xiii) relationship strength; (xiv) issue awareness; and (xv) cost savings (Table 8). 

*Health status*, including well-being and quality of life, understood as physical, mental and social health and well-being, have been found to be the most measured outcomes in the healthcare sector. These outcomes have been mainly assessed with recognised, validated, self-reported scaled and clinical objective indicators, such as blood biochemical parameters [104]. The most recurrent metrics are the Patient Health Questionnaire (PHQ-9, [105]) (e.g., [18]); Health of the Nation Outcome Scales (HoNOS, [106]) (e.g., [63,107]); and Warwick–Edinburgh Mental Well-being Scale (WEMWBS, [108]) (e.g., [109]). Given the scarce investigation of informal caregiver perspectives, it is interesting to mention the study of Wood et al. (2010) [110] that used the Quality of Life Questionnaire for Family (QLQ-F; [111]) and treatment Group (QLQ-G, [111]) for assessing the perceived quality of family life after co-created training for people with addiction disorder and their caregivers.

*Satisfaction* includes all the metrics assessing the lay actor’s subjective perception of co-produced service experiences, outcomes and processes. Satisfaction with a service (mostly derived from performance and service outcomes) and with the process (mainly referring to the enjoyment of collaboration) have been considered. These dimensions have been measured both with validated scales and ad hoc or adapted scales, whose reliability and validity are generally verified in studies. Within the first tools, the Social Support Programme Acceptability Rating Scale ([112]) was used by Brown et al. (2020) [18] both to evaluate the acceptability (outcomes for provider) and satisfaction of a participant in community co-produced intervention. Ad hoc or adapted instruments draw on some key previous studies, such as the perceived value scale developed in Sweeney and Soutar (2001) [21] or the service quality scale developed in Dagger et al. (2007) [113]. Hau (2018) [43] measured outcome (perceived) values and process (perceived) value in health co-creation with Hau and Thuy’s (2012) scales [114]. In the public domain, Sanina et al. (2020) [66] used an ad hoc scale for assessing students’ satisfaction with the co-produced activities in which they were involved. Within metrics used to assess informal caregivers’ satisfaction, the “adjusted version of the caregivers’ satisfaction with inpatient stroke care (C-SASC, [115]) 11-item scale” [116] and Family Experiences with Coordination of Care (FECC, [117]) Measure Set [77] have been used.

*Activation* defines one’s ability (knowledge, skills and confidence) to take independent actions in one’s life, for example to manage their care. In the health domain, this dimension is often measured with the well-known Patient Activation Measures (PAM, [118]), such as in Turner et al. (2015) [119], which used this scale to assess the effect of co-produced self-management training. The activation in the health field has been mostly interpreted as confidence in self-management [119,120], while in the public sector it has been assessed as the level of activeness regarding participation [121]. 

It should be emphasised that the PAM has also been used for assessing perceived *empowerment*, as in Jo and Nabatchi, 2019 [99]. It could be interpreted as a status (or) process by which people gain control over their lives. Empowerment dimension includes metrics that assess lay actors’ capacities to become co-producers of health and well-being for their own [99] or for their loved ones [109,122]. The empowerment dimension in the public sector has also been measured with an adapted scale, such as the Self-Report Level of Participation Survey, which aims at assessing the perceived sense of involvement of lay actors during co-design workshops [123]. 

*Self-management* has been defined as the confidence of patients in self-care. It has been assessed through validated scales, such as the Health Education Impact Questionnaire (heiQ, [124]) (e.g., [119,120]) and Summary of Diabetes Self-Care Activities ([125] in [126]). Other psychological benefits regarding *self-efficacy, self-esteem* and *self-confidence* have rarely been assessed through metrics within the healthcare and public sectors. Fors and colleagues [50,127,128] evaluated in a randomised control trial the effects of person-centred and co-created care in acute disease using the General Self-Efficacy Scale (GSE scale, [129]); Wood et al. (2010) [110] used Rosenberg Self-Esteem Scale (RSE, [130]), while, in the public sector, Sanina et al. (2020) studied self-efficacy by looking at students’ confidence in their professional skills [66]. 

The positive and negative effects of co-production on stress have been assessed regarding *eustress* and *burden* with validated or adapted instruments in the healthcare field, such as the Burden Assessment Scale [131], which was used in Chiocchi et al. (2019) [109] to evaluate the effect of co-delivery psychoeducation programme on carer burden.

Both in the public and health academic literature, *learning and behaviour change* have been widely recognised as a possible effect of co-production. In quantitative terms, learning outcomes have been assessed regarding health literacy with validated (i.e., the newest Vital Sign UK (NVS-UK, [132]) ([18]), Diabetes Knowledge Test (DKT, [133]) in New et al. (2010) [126]) or ad hoc scales, and also with specific indicators, such as the learning scores of teaching tests [66,134]. Other behaviour/attitude changes include (improved) compliance, explained as the extent to which patients (or other lay actors) follow service provider’s instructions, decisional conflict, that is, a state of uncertainty about the actions to be taken or opinions to be expressed, or a change in life routine (e.g., eating style). They have been assessed mainly in the health domain, with a validated scale or ad hoc adapted instruments (e.g., [81,94]). Some indicators are also adopted (e.g., [72,135]). Even though the change of behaviour or attitude has been mainly assessed in the health sector, the ad hoc scale suggested by Chen et al. (2015) deserves attention since it also investigates possible negative changes of behaviours in lay actors, such as unethical and rude behaviours [100].

The last three dimensions are few and measured largely with ad hoc instruments.

*Relationship strength* concerns a better relationship with professionals; the bidirectional value of this outcome means that it is also used for lay actors. It has been measured with an ad hoc or adapted instrument, as already seen in the sub-paragraph on professionals’ outcomes. 

*Issues awareness* is only investigated in Jo and Nabatchi (2019) by measuring people’s perception of the importance of a given issue with an ad hoc item [99].

*Cost saving* is mainly discussed in relation to providers; however, some authors also highlight that there are cost effects for lay actors. For instance, in their cost minimisation analysis, Spanò et al. (2018) also consider lay actors’ perspectives, which include missed work/school days and travel expenses for visits [16].

#### 3.3.4. Outcome for Community

The metrics that assess outcomes on the community are the fewest. They have been classified into (i) value for the community and (ii) value for society (Table 9). No metrics have been identified for assessing the third category devised by Cepiku et al. (2020), socioeconomic impact [29].

*Value for community* has been defined as increasing trust towards service providers and the better understanding of service costs and procedures thanks to direct collaboration with citizens [152]. Although the co-production literature often refers to this concept in explaining outcomes of co-production [2], it is recognised as difficult to define and, especially, to measure. Indeed, only a few studies have used quantitative metrics for assessing value for community regarding social capital. These studies mainly refer to community co-production, for instance in public health interventions, such as Bolton et al. (2016) and Brown et al. (2020), who adopted the Adapted Social Capital Questionnaire [153] and Arizona Social Support Interview Schedule [154], respectively [18,48].

*Value for society* has been defined as the result of three co-production effects: democratisation of the process, equal distribution of effects on society and increasing public acceptance [29]. The equal distribution of co-production effects on society has been measured with a validated scale, adapted instrument, ad hoc items and indicators. More precisely, it assesses the reduction of health or other public services, for example inequalities in deprived areas or poor countries, for instance, regarding the increasing number of service participants [20,76], the increasing identification of children’s needs (National Survey of Children with Special Health Care Needs [155]) and the increasing quality and accessibility of public commodities such as water [64]. 

In assessing the effects on the community, the public findings bring important insights to the health field, especially in measuring the value for society. While health findings focus on effects related to community health and well-being [77], public ones broaden the perspective by investigating the accessibility and economic affordability of the service and environmental effects.

## 4. Discussion

The review confirms that healthcare is a sector where the interest in co-production evaluation is significantly increasing [1,2,3]. Health is also a highly investigated field among public sector scholars, where the urgency of focusing a co-production research agenda on outcome evaluation is widely recognised [15]. 

Despite this increasing attention of academics and practitioners, the literature on co-production empirical studies returned a fragmented picture regarding study design (defining “when” co-production outcomes are assessed), approaches, methods and tools (identifying “how” is measured) and specific metrics adopted (“what” is measured). 

Considering the research design, less than a quarter of the included studies are based on longitudinal or mixed design (i.e., cross-sectional and longitudinal). It implies that most target the hic and nunc relationships between features of co-production and outcomes, without comparing groups (e.g., co-producers vs. non-co-producers), and ex ante and ex post benchmarks or how outcomes evolve over time (i.e., co-production sustainability). The adoption of a comparative research design would help to enforce the robustness and sustainability of the results of the evaluation effort. 

Moreover, the analysis of the research approach reveals the clear predominance of qualitative studies, while the use of quantitative or mixed methods is limited to a quarter of the included papers. Considering the complexity of the topic and its multi-stakeholder and intrinsically multilevel nature, the combination of quantitative and qualitative approaches would make its understanding more robust and valid [157].

Delving deeper into the quantitative approach, findings present a wide range of tools and metrics (i.e., internationally validated scales, other reliable tools, adapted or ad hoc instruments, or single indicators), used for the evaluation of co-production. The study also provides their classification by specific outcome and actor. The paper offers a multidimensional measurement system that researchers and/or managers can use by choosing the most suitable tools, according to the specific measurement needs (e.g., type of co-production, context, actor). For instance, in the case of e-health co-design, as in theextant literature (e.g., [69]), it may be useful to use the SUS scale and measure the acceptability of the co-created e-tool. Metrics aimed at measuring changes in skills and personal behaviour in clinical routines, such as professionals’ empathy or attitude towards patients, may help to evaluate the outcomes on co-delivery training (as in [93]). Furthermore, the measure of improvement (or worsening) of health status has often been used in assessing the effects of a co-delivered health service (e.g., [50]); however, the choice of the most appropriate measure should also take into account the specific health context/disease of the patient (e.g., diabetes, motor difficulties, mental disability, etc.). 

Currently, some important differences have emerged throughout the actors considered (i.e., regular providers, professionals, lay actors and community) [29].

Specifically, the use of validated tools, borrowed from medicine (e.g., General Health Questionnaire-12 [48]), health psychology (e.g., Hospital Anxiety and Depression Scale (HADS) [119,120,136,137]) and organisational well-being studies (e.g., Social production function scale (SPF-IL) [46,88]), is more widely adopted to evaluate the impacts on lay actors and professionals. Recognised and validated constructs and scales have been used mainly in experimental health research and health service management research, while scarce contributions originated from the public sector. Such results could be fascinating for scholars investigating impacts for users of public service and public officials. 

Looking closely at outcome dimensions, unsurprisingly, lay actors are the perspective that captured the main attention of scholars in the health field, with almost half of the retrieved outcomes. The number of sub-dimensions (15) also reflects the maturity of evaluation on this topic. Health status, well-being and quality of life are largely the dimensions that offer a portfolio of validated scales (e.g., health status: Patient Health Questionnaire (PHQ-9) [18]; well-being: Warwick-Edinburgh Mental Well-being Scale (WEMWBS); [48,109,135,136]; quality of life: EuroQol index (EQ 5D index) [119,120]), followed by satisfaction with service (e.g., Psychological Needs Satisfaction in Exercise Scale [49]). Interestingly, some well-known constructs in medicine and health psychology (such as enjoyment [43], self-management [119,120], self-efficacy [50], self-esteem [110], eustress [23], burden [109], trust [73] and issue awareness [99]) have been relatively scarcely analysed. 

Overall, the review returns a paucity of attention to a key player in health co-production: the informal caregiver (e.g., [77,110,116]). Given the scant investigation of informal caregiver perspectives, further research is needed to explore the outcome dimensions related to the stakeholders and the research approach that best can evaluate their role. Helping with the daily management of an illness causes caregivers to display a predictable decrease in psycho-physical well-being; indeed, the literature describes them as “hidden patients” [158]. As claimed by Ens et al., (2014), the consequences of caregivers’ inadequate support negatively influence not only the patient, but also the whole healthcare system (e.g., may cause a greater dependency on institutional healthcare providers and increase medical visits needs, also impacting overall healthcare costs) [159]. Thus, the inadequacy of the caregiver could also act as a deterrent in co-producing. 

Last, it is interesting to call attention to the ad hoc scale suggested by Chen et al., (2015) in the public realm, which also aims at explicitly investigating possible negative changes of behaviours in lay actors [100].

The regular provider perspective is the second-most investigated outcome after lay actors. It has been mainly investigated through ad hoc tools or measures, except cost savings (thanks to the well-developed cost–benefits analysis [160]) and more technical service conditions (such as usability and acceptability/feasibility, e.g., [48,69,71,81,82]). In this domain, the public sector literature offers interesting hints with regard to the service improvement dimension (assessing frequency, distance from users [64] rather than the mere number of service users involved [20,76]) and the reorganisation of the government structure regarding the decentralisation of power [76].

Only 17% of the investigated outcomes considered the professionals’ perspective. Staff well-being and empathy/attitude are more widely analysed (e.g., [46,88,93,94,101]), thanks to well-known international scales targeting these issues. Public metrics assessing the type of outcome are limited in number and do not add valuable and innovative insights to health findings (e.g., [100]). 

Finally, in assessing outcomes on community, the contribution from the health literature is still limited (e.g., [18,77]). The public findings bring important insights to the health field, especially in measuring the value for society. While health findings focus mainly on effects related to community health and well-being [77], public ones broaden the perspective by examining the accessibility [76,156] and economic affordability of the service [64] and environmental effects [80]. 

## 5. Conclusions

The results presented and discussed in this article provide a blueprint co-production multidimensional performance measurement system that factors in the values and perspectives of multiple stakeholders. The coding framework has been structured according to the outcome dimensions in the co-production literature [15,29], adapting the analytical components concerning health specificities. In this way, the findings guide the further development of a theoretical framework of value co-production. The paper offers a systematisation of the methods, tools and metrics used to assess outcomes of co-production. Thus far, studies have discussed the effects of co-production, but they have not focused on how these outcomes have been evaluated. Moreover, the framework developed in this paper identifies a new stakeholder dimension: professionals. The role of the workforce is particularly interesting to evaluate separately from the regular provider, as a required specific approach. 

This framework can be used in other contexts with cross-sector adaptability. Health emerged as a widely investigated field for the assessment of co-production; this health literature embraces both public and private streams. Health resulted in the top investigated domain in co-production public management; moreover, the service management literature has increasingly focused on the healthcare service setting, considered a rich and fertile context in which to explore a new service delivery model due to its uncommon and complex characteristics [27]. 

Thus, the findings offer a blueprint multidimensional quantitative performance measurement system that can inform evaluation co-production academic research across research fields, healthcare, public and service management. 

The results offer a systematic overview of the literature focusing on co-production evaluation in health, providing performance measurement system tools and measures available and tested in the literature for each sub-dimension. These indications help to strengthen the understanding and analysis of co-production outcomes. The performance system promotes improvements in empirical data collection on the multiple faceted co-produced activities and spurs consciousness of the adoption of sustainable patient-based initiatives. 

This paper offers the opportunity to develop a multi-metric evaluation study. The results showed that the current research is mainly focused on mono-stakeholder impact. This study provides the opportunity to design a configurational approach that goes beyond the focus on a single outcome to illuminate how multiple outcomes interact and interrelate. Outcomes can self-reinforce each other (e.g., the enhancement in professional motivation and patient engagement) or manifest trade-offs (e.g., provider costs or efforts and patient satisfaction). 

Finally, the paper offers insights for managers and community leaders engaged in co-production initiatives, supplying a conceptual classification of several impacts affecting co-production and an operational guide to better design and implement an empirical multi-dimensional performance management system. COVID-19 has forced an increase in the co-production of services in the public and health sectors [11]. This trend will be consolidated to boost the development of new co-production practices that will require a robust and replicable measurement and evaluation system. 

Noticeably, the paper has also revealed five critical research gaps that could inform the further research agenda on co-production. First, there is a lack of consideration of the impact of the co-production process on informal caregivers and their crucial role in sustaining or interfering with patients’ behaviours and motivation to co-produce. Secondly, another understudied area is the impact of co-production on professionals. As workers’ wellbeing is an important quality indicator in the delivery of high-quality care, it is important to investigate if and how co-producing with the patient can lead to an increase or decrease in the professionals’ wellbeing, including relevant individual and organisational variables. Third, community is not only a neglected outcome dimension, but it also suffers from weaker methodological approaches. Furthermore, the literature still uncovers unfavourable effects of co-production across the multi-stakeholder outcome dimension. Last, the findings still show a lack of empirical mixed-method approaches applied to co-production, which could enhance with robust and valid insights the understanding of how co-produced service affects various stakeholders. A deeper investigation of the type and robustness of qualitative approaches could help to select a more suitable research tool to address the evaluation effort. Moreover, a longitudinal perspective would strengthen the understanding of how co-production impacts unfold over time, addressing the sustainability challenge.

Although the paper aims to be as comprehensive and replicable as possible, it contains some limitations, mostly directly related to the systematic review method. The choice of keywords and the formulation of the query used in search strategy may have resulted in some eligible studies being undetected because they used a different terminology. However, including both co-production and co-creation, which are often used interchangeably [33,34], as well as resorting to the snowball technique, has helped to mitigate this issue. The second limitation is caused by the inclusion of only peer-reviewed journal publications in English. Despite this choice ensuring a scientific and methodological rigour of the findings, it neglected books, conference papers, grey literature and works published in other languages which could have added other interesting insights. Finally, some evaluation approach may be missed because the paper aims at collecting empirical evidence and the theoretical papers and protocols were excluded. 

Despite these limitations, the review presents points of originality and was carried out on a large number of studies with a transparent and well-documented process. It helps to advance knowledge of co-production and supports researchers and practitioners in future endeavours. 

## Figures and Tables

**Table 1:** Search query structure.

Levels	Keywords_Health Dataset	Keywords_Public Dataset
**First level** **(AND)**	Co-product* OR Co-creat* OR Coprod* OR Cocreat*	Co-product* OR Co-creat* OR Coprod* OR Cocreat*
**Second level** **(AND)**	Health*	“Public Servic*” OR “Public Sector*”
**Third level**	Evaluat* OR Impact* OR Assess* OR Outcome* OR Indicator* OR Measur*	Evaluat* OR Impact* OR Assess* OR Outcome* OR Indicator* OR Measur*

**Table 2:** Studies’ contexts in the healthcare domain.

Context		n. Papers
**Chronic or** **long-term conditions**	Mental health, Elder care, Cancer, Diabetes, Heart disease, Rare chronic disease	100 (11 on e-health and 2 on assistive technology)
**Public health**	Health prevention and promotion, Health inequalities (cancer screening, maternity, mental health, healthy lifestyle, palliative care)	23 (2 on e-health)
**Acute conditions**	Osteoarthritis, Acute coronary syndrome, Self-harm	6 (1 on e-health)
**Others**	Integrated care, Primary care, Secondary care, Homecare in rural areas, Multiple settings	13 (2 on e-health)
**Not specified**		19 (3 on e-health)

**Table 3:** Research approach of reviewed articles.

Research Approach	Total Dataset		Healthcare		Public	
	n.	%	n.	%	n.	%
**Qualitative**	96	47.3	72	44.7	24	57.1
**Quantitative**	54	26.6	47	29.2	7	16.7
**Mixed Methods**	53	26.1	42	26.1	11	26.2
**Total**	**203**	**100**	**161**	**100**	**42**	**100**

**Table 4:** Mono-stakeholder/multi-stakeholder evaluation approach.

	Mono-Stakeholder	Multi-Stakeholder
**Health**	47.8%	52.2%
**Public**	42.9%	57.1%

**Table 5:** Methods for stakeholder outcome evaluation.

Methods of Data Analysis	Outcome for Provider			Outcome for Professionals			Outcome for Lay Actors			Outcome for Community		
	*H%*	*P%*	*T%*	*H%*	*P%*	*T%*	*H%*	*P%*	*T%*	*H%*	*P%*	*T%*
**Total**	32	43	**35**	18	15	**17**	44	31	**41**	6	11	**7**
**Quantitative**	*46*	*30*	*41*	*29*	*10*	*26*	*46*	*38*	*45*	*24*	*18*	*22*
**Qualitative**	*54*	*70*	*59*	*71*	*90*	*74*	*54*	*62*	*55*	*76*	*82*	*78*

H% refers to health publications only; P% refers to public publications only; T% refers to the overall publications. In bold, the total outcome per stakeholder.

**Table 6:** Measures and metrics of outcomes for provider.

Outcome		International Validated Scales	Other Tools	Indicators	Main References
**Cost-efficiency**	*Cost savings*		Cost—benefits analysis (cost-minimization analysis [16]; opportunity-cost analysis) [17];	Length of admission x average costs per night [62];	[16,17,62];
	*Efficiency*			Reductions of: occupied bed days; hospital admission; days on a community treatment and community contacts [63];	[63];
**Effectiveness**	*Service* *improvement*		Ad hoc [64,78]*;	- N. person using the service [76] *; - N. persons screened/target [20,72]; - Offering a specific type of service or not [65]; - Students’ examination results and game scores [66,67] *; - % of recycling on the total amount of waste achieved by each municipality [79] *; - Count variable calculating by summing the pro-environmental activities undertaken [80] *;	[20], [64] *, [65], [66] *, [67] *, [72]; [76] *, [78] *, [79] *, [80] *;
	*Usability of* *e-tools*	System usability scale (SUS) [69,81,82];	Ad hoc [83], [84] *;	N. of user requests for service support [84] *;	[69,81,82,83,84] *;
	*Acceptability/* *feasibility of* *interventions/* *e-tools*	- Social Support Programme Acceptability Rating Scale [48]; - Patient Education Materials Assessment Tool (PEMAT) [71]; - Training Acceptability Rating Scale (TARS) [52];	Ad hoc [85,86];	N. or % of patients declining (or accepting) the intervention [72];	[48,52,71,72,85,86];
**Trust**			Ad hoc [73], [74] *;		[73], [74] *;
**Loyalty**			Ad hoc [73];		[73];
**Behavioural** **intentions**			- Construct of Dagger et al. (2007) [45,75]; - Construct of Zeithaml et al. 1996 [21]; - Ad hoc [66,78] *, [23,43,87]		[21,23,43,45], [66] *, [75], [78] *, [87];
**Innovation**			Ad hoc [42];		[42];
**Adaptability**/ **Flexibility**		CSHCN Stand-Alone Questionnaire [77];	Adaptation from Voorberg et al. 2015 [76] *;		[76] *, [77];

The asterisk * indicates publications in the public domain.

**Table 7:** Measures and metrics of outcomes for professionals.

Outcome		International Validated Scales	Other Tools	Main References
**Job** **satisfaction**		Measurement of Job Satisfaction (MJS) [46,88];	Ad hoc [6,89];	[6,46,88,89];
**Staff** **Well-being** **(including burnout)**		- Social production function scale (SPF-IL) [46,88]; - Maslach Burnout Inventory [93,94];		[46,88,93,94];
**Work** **engagement**		Utrecht Work Engagement Scale (UWES) [6];	Co-productive-taxpayer-as-supervisor (CTS) construct [100] *;	[6], [100] *;
**Motivation**		Staff Positive Contributions Questionnaire (short version) [97];		[97];
**Behavioural change**	*Helping* *behaviour*		Adapted from Schneider et al. 2005 [6];	[6];
	*Empathy/* *Attitudes*	- Personality disorder-knowledge, Attitudes and Skills Questionnaire [47,93,101]; - Similarities and empowerment attitude sub-scales from the Community Living Attitudes Scale [97]; - Staff Empathy for people with Challenging Behaviour Questionnaire (SECBQ) [97]; - The borderline personality disorder-cognitive/emotional attitudes inventory (BPD-CAI/FAI) [98];	Attitude towards self-harm in CYP of Crawford et al. (2003) [102];	[47,93,97,98,101,102];
	*Learning*	- Personality Disorder-Knowledge, Attitudes and Skills Questionnaire [47,93,101]; - Borderline personality disorder- cognitive/emotional attitudes inventory (BPD-CAI/FAI) [98];	Adapted from Crawford et al. (2003) [103];	[47,93,98,101,103];
	*Self-* *efficacy*		- Challenging Behaviour Self-Efficacy Scale (CBSE) [97]; - Adapted version of the Self-efficacy Towards Helping Scale [103];	[97,103];
	*Clinical* *behaviour* *intentions*	Continuing Professional Development Reaction Questionnaire [103];		[103];
	*Confidence*		Ad hoc [103];	[103];
**Relationship-strength**			- Adapted from Hall et al., 2001 [99]; - Adapted from Rajah et al. (2008) [73];	[73,99];

The asterisk * indicates publications in public domain.

**Table 8:** Measures and metrics of outcomes for lay actors.

Outcome		International Validated Scales	Other Tools	Indicators	Main References
**Health status, well-being and quality of life**	*Physical and* *mental health* *status*	- General Health Questionnaire-12 [48]; - Health of the Nation Outcome Scales HoNOS [63,107]; - Generalized Anxiety Disorder Questionnaire (GAD-7) [18]; - Patient Health Questionnaire (PHQ-9) [18]; - Hospital Anxiety and Depression Scale (HADS) [119,120,136,137]; - EORTC QLQ-C30 [137]; - Functional Assessment of Chronic Illness Therapy—Fatigue FACIT-F [137]; - Insomnia Severity Index (ISI) [137]; - Saltin Grimby Physical Activity Level Scale (SGPALS) [50]; - Strengths and Difficulties Questionnaire [86,138]; - Post-Traumatic Stress Disorder (PCL-C); [137]; - Mood andFeelings Questionnaire (MFQ) [86]; - Revised Child Anxiety and Depression Scale (RCADS) [86]; - Western Ontario and McMaster University Osteoarthritis Index (WOMAC) [139];	Ad hoc [140];	- Improved/deteriorated or unchanged clinical indicators (e.g., waist-to-height ratio; carotid artery reactivity (CAR); blood biochemical parameters; death) [49,104,136]; - Re-admission/re-hospitalization for unscheduled reasons; occurrence of an adverse outcome [50,141];	[16,18,21,22, 44,45,48,49,50,51,63,86,104,107,109,110,116,119,120,135,136,137,138,139,140,141,142,143];
	*Quality of life and health-related quality of life*	- Quality Quality-Adjusted Life-Years (QALYs) using HAE-BOIS-Europe survey [16]; - EuroQol index (EQ 5D index) and the EuroQol Visual Analogue Scale (EQ VAS) [119,120]; - Quality of Life Questionnaire for family (QLQ-F) and treatment group (QLQ-G) [110];	Fox Simple Quality-of-Life Scale [21];		
	*Well-being*	- 15-item version ofthe Social Production Function Instrument for the Level of Well-being (SPF-ILs) (both patients and carers); [51,116,142,143]; - Warwick-Edinburgh Mental Well-being Scale (WEMWBS); [48,109,135,136]; - McGill Quality of Life Questionnaire (MQOL index) [44]; - McGill quality of life questionnaire (MQOL index)-revised [22];	Perceived well-being construct of Sweeney et al. (2015) [144];		
**Satisfaction**	*With service/care*	- Social Support Programme Acceptability Rating Scale [48]; - Patient Assessment of Chronic Illness Care Short version (PACIC-S) [143]; - Psychological Needs Satisfaction in Exercise Scale [49]; - Family Experiences with Coordination of Care (FECC) Measure Set [77]; - Diabetes Management and Evaluation Tool (DMET) [126];	- Perceived service quality and satisfaction construct of Dagger et al., 2007 [45,75]; - Perceived quality of service questionnaires of Veltro et al. (2007) [107]; - Service satisfaction scale of Oliver 2010 [21]; - Outcome value scale of Hau and Thuy, 2012 [43]; - Adjusted version of the caregivers’ satisfaction with inpatient stroke care (C-SASC) 11-item scale [116]; - Adjusted version of the Satisfaction with Stroke Care questionnaire (SASC) [51,116,142]; Adaptation from: - Rajah et al., 2008 [73]; - Aiken and Patrician (2000) [94]; - Yu and Dean (2001) [145]; - Wang et al. (2004) and Sweeney and (2001) [145]; - Sweeney and Soutar (2001) and Mathwick et al. (2001) [146]; - Sweeney and Soutar (2001) [75,147]; - Suarez-Alvarez et al. (2020) [148]; - Ad Hoc [64,66,74] *, [6];		[6,21,43,45,48,49,51], [64] *, [66] *, [73], [74] *, [75,77,94,107,116,126,142,143,145,146,148]
	*With process* *(enjoyment)*		- Process value of Hau and Thuy, 2012 [43]; Adaptation from: - Wang et al. (2004) and Sweeney and Soutar (2001) [145,147]; - Nelson and Byus (2002) [75]; - Ad hoc [144];		[43,45,75,145,147]
**Activation**		Patient Activation Measure [119,120,135]	Ad hoc [121] *;		[119,120], [121] *, [135]
**Empowerment**		- Family Empowerment Scale [77,109] - Patient Activation Measure (PAM) [99]; - SIS Communication/Behavior Skills Questionnaire [110]; - Diabetes Empowerment Scale Short Form (DES-SF) [126];	- Adaptation from spidergram tool of Draper et al. (2010) [123] *; - Ad hoc [122] *;		[77,99,109,110], [122] *, [123] *, [126]
**Self-** **management**		- Health Education Impact Questionnaire (heiQ) [119,120]; - Summary of Diabetes Self-Care Activities (DSCA) [126];			[119,120,126]
**Self-** **efficacy**		General Self-Efficacy Scale (GSE scale) [50];	Ad hoc [66] *;		[50], [66] *;
**Self-** **esteem**		Rosenberg Self-Esteem Scale [110];			[110];
**Self-** **confidence**			Ad hoc [149];		[149];
**Eustress**			Adaptation from: Eustress construct of Simmons and Nelson 2001/Nelson and Simmons 2003 [23];		[23];
**Burden**		Burden Assessment Scale (BAS) [109];	Family worry of Center for Medical Home Improvement [77];		[77,109]
**Learning and change** **behaviour/** **attitude**	*Health literacy and knowledge*	- Newest Vital Sign UK (NVS-UK) [18]; - Diabetes Knowledge Test (DKT) [126];	Ad hoc [81,149];	Score of learning test [66,134] *;	[18], [66] *, [81,126], [134] *, [149]
	*Compliance/* *adherence*		- Adaptation from Hausman 2004 [23,146]; - Ad hoc [23];	Tracking completion of indicated quarterly follow-up screens [72];	[23,72,146];
	*Decisional conflict/* *self-determination/* *self-advocacy*	- Decisional Conflict Scale (dcs) [81]; - SURE (Sure of myself, Understand information, Risk–benefit ratio, Encouragement) [81]; - Self-Determination Theory (SDT) scales [150];	Adaptation from: - Behavioural Regulation in Exercise Questionnaire [151]; - Patient Self-Advocacy Scale [149];		[81,149,150,151]
	*Others*	- Recovery Attitudes Questionnaire7 (RAQ-7) [149]; - ATT-19 [126]; - Physical Activity Questionnaire for adolescent [138];	- Unhealthy eating adapted from Stansfeld et al., 2003 [138]; - Ad hoc [66,100] *, [138,149];	- N. of consultations offered and attended were measured by exercise referral practitioners [49,126]; - Job opportunities after or during recovery [135];	[49], [66] *, [100] *, [126,135,138,149]
**Relationship strengths with** **professionals**			Adaptation from Rajah et al., 2008 [73];		[73]
**Issue** **awareness**			Ad hoc [99];		[99]
**Cost** **savings**			Cost minimization analysis [16]; Ad hoc health expenses [140];		[16,140]

The asterisk * indicates publications in public domain.

**Table 9:** Measures and metrics of outcomes for community.

Outcome		International Validated Scales	Other Tools	Indicators	Main References
**Value for community**	*Social* *capital*	Arizona Social Support Interview Schedule (ASSIS) [18]	Adapted Social Capital Questionnaire [48]		[18,48]
**Value for society**	*Access to* *services/* *democratization*	National Survey of Children with Special Health Care Needs [77];	Ad hoc [64] *	- % Increase in participation in the screening program with vulnerable population [20]; - Increase access to health or public services [76,156] *; - Count variable calculating by summing the pro-environmental activities undertaken [80] *	[20], [64] *, [76] *, [77], [80] *, [156]

The asterisk * indicates publications in public domain.

## Data Availability

Data are available on request.

## Supplementary Materials

The following are available online at https://www.mdpi.com/1660-4601/18/7/3336/s1. Table S1: Main journal and subject area of reviewed articles; Table S2: Most analysed countries in the reviewed articles; Table S3: Study context in public domain.
