# Supplementary material for: Co-Production Performance Evaluation in Healthcare. A Systematic Review of Methods, Tools and Metrics
Source: Int J Environ Res Public Health. 2021 Mar 24;18(7):3336. doi: 10.3390/ijerph18073336 (PMC8037812; doi:10.3390/ijerph18073336)
Supplement: Supplementary file 1 [file ijerph-18-03336-s001.zip › Supplementary materials/Supplementary material 1_Journal and disciplines of reviewed articles.docx]

*Table S1a: Main journal and subject area of reviewed articles in healthcare dataset*

| Journal | Published papers | H- index | Subject area (and category) |
| --- | --- | --- | --- |
| *Mental Health And Social Inclusion* | 8 | 11 | Nursing *(Psychiatric Mental Health)*; Medicine *(Psychiatry and Mental Health)*; Social Science *(Health)* |
| *BMJ Open* | 6 | 84 | Medicine (*miscellaneous)* |
| *Health & Social Care In The Community* | 6 | 65 | Social science (*Miscellaneous; Social Work; Sociology and Political Science*); Medicine *(Health policy; Public Health, Environmental and Occupational Health)* |
| *International Journal Of Environmental Research And Public Health* | 6 | 92 | Medicine (*Public Health, Environmental and Occupational Health*); Environmental Science (*Health, Toxicology and Mutagenesis; Pollution*) |
| *Journal Of Mental Health Training, Education And Practice* | 5 | 13 | Medicine *(Health Policy; Psychiatry and Mental Health*); Business, Management and Accounting (*Organizational Behavior and Human Resource Management*); Social Science (*Education; Health*); Nursing (*Psychiatric Mental Health*) |
| *JMIR Mhealth And Uhealth* | 4 | 12 | Medicine (*Health Informatics*) |
| *Journal Of Psychiatric And Mental Health Nursing* | 4 | 60 | Nursing (*Psychiatric Mental Health*) |
| *Journal Of Service Theory And Practice* | 4 | 79 | Business, Management and Accounting (*Strategy and Management*) |
| *Patient Education And Counseling* | 4 | 131 | Medicine (*miscellaneous*) |
| *Service Business* | 4 | 28 | Business, Management and Accounting (*Business and International Management; Strategy and Management*) |
| *Bmc Health Services Research* | 3 | 101 | Medicine (*Health Policy*) |
| *Health Expectation* | 3 | 68 | Medicine (*Public Health, Environmental and Occupational Health*) |
| *International Journal Of Mental Health Nursing* | 3 | 50 | Nursing (*Psychiatric Mental Health*) |
| *Journal Of Medical Internet Research* | 3 | 127 | Medicine (*Health Informatics*) |
| *Journal Of Service Research* | 3 | 113 | Business, Management and Accounting (*Organizational Behavior and Human Resource Management)*; Computer Science (*Information Systems*); Social Science (*Sociology and Political Science*) |
| *Journal Of Services Marketing* | 3 | 96 | Business, Management and Accounting (*Marketing*) |
| *Public Management Review* | 3 | 60 | Business, Management and Accounting *(Management Information Systems; Management of Technology and Innovation)*; Social Science (*Public Administration*) |

*Table S1b: Main journal and subject area of reviewed articles in public dataset*

| Journals | Published papers | H-index | Subject area (and category) |
| --- | --- | --- | --- |
| *Public Management Review* | 5 | 60 | Business, Management and Accounting (*Management Information Systems; Management of Technology and Innovation*); Social Science (*Public Administration*) |
| *Public Money and Management* | 3 | 56 | Business, Management and Accounting (miscellaneous); Social Sciences (Public Administration; Sociology and Political Science) |
| *International Journal of Public Administration* | 2 | 41 | Business, Management and Accounting (*Business and International Management*); Social Sciences (Public Administration) |
