# Supplementary material for: Co-Production Performance Evaluation in Healthcare. A Systematic Review of Methods, Tools and Metrics
Source: Int J Environ Res Public Health. 2021 Mar 24;18(7):3336. doi: 10.3390/ijerph18073336 (PMC8037812; doi:10.3390/ijerph18073336)
Supplement: Supplementary file 1 [file ijerph-18-03336-s001.zip › Supplementary materials/Supplementary material 2_Most investigated countries.docx]

*Table S2: Most analysed countries in the reviewed articles*

| Healthcare | | | Public | | |
| --- | --- | --- | --- | --- | --- |
| Top 5 countries | **n.** | **%** | **Top 5 countries** | **n.** | **%** |
| UK | 71 | 44,10 | UK | 10 | 23,81 |
| USA | 15 | 9,32 | Netherlands | 5 | 11,90 |
| Netherlands | 13 | 8,07 | Denmark | 4 | 9,52 |
| Australia | 10 | 6,21 | Italy | 4 | 9,52 |
| Italy | 8 | 4,97 | Finland | 3 | 7,14 |
| Total | **117** | **72,67** | **Total** | **26** | **61,90** |
