# Supplementary material for: Co-Production Performance Evaluation in Healthcare. A Systematic Review of Methods, Tools and Metrics
Source: Int J Environ Res Public Health. 2021 Mar 24;18(7):3336. doi: 10.3390/ijerph18073336 (PMC8037812; doi:10.3390/ijerph18073336)
Supplement: Supplementary file 1 [file ijerph-18-03336-s001.zip › Supplementary materials/Supplementary material 3_Field in public domain.docx]

Table S3: Study context in public domain

| Fields | N. paper |
| --- | --- |
| Health & social care | 3 (+ 8)* |
| Education | 10 |
| Government – general | 8 |
| E-governance | 5 |
| Environment | 3 |
| Water management | 3 |
| Justice and security | 3 |
| NGO (rural service/social care) | 2 |
| Neighbourhood | 2 |
| Infrastructure | 1 |
| Entrepreneurship | 1 |

* 4 papers are included in public dataset, 8 were in overlapping with healthcare dataset and they are included only in those dataset.
